# Closing the Gaps in Heart Failure Care: A Call for a National Strategy in Canada

**DOI:** 10.1016/j.cjco.2026.07.002

**Published:** 2026-07-08

**Authors:** Stephanie Poon, Sean A. Virani, Margot K. Davis, Santabhanu Chakrabarti, Anique Ducharme, Lynette Kosar, Robert S. McKelvie, Nima Moghaddam, Seema Nagpal, Lebei Pi, Rodolfo Pike, Anne Simard, Harindra Wijeysundera

**Affiliations:** a Division of Cardiology, Sunnybrook Health Sciences Centre, University of Toronto, Toronto, Ontario, Canada; b Division of Cardiology, University of British Columbia, Vancouver, British Columbia, Canada; c Division of Cardiology, University of Montréal, Montréal, Québec, Canada; d Outpatient Cardiology Services, Saskatchewan Health Authority and College of Pharmacy, University of Saskatchewan, Saskatoon, Saskatchewan, Canada; e Division of Cardiology, University of Western Ontario, London, Ontario, Canada; f Canadian Cardiovascular Society, Ottawa, Ontario, Canada; g Division of Cardiology, Mackenzie Health, Toronto, Ontario, Canada; h Newfoundland and Labrador Health Services, St. John's, Canada; i Peter Munk Cardiac Centre, University Health Network, Toronto, Ontario, Canada

**Keywords:** equity, health policy, heart failure

## Abstract

Heart failure (HF) is a growing public health challenge in Canada, affecting over 787,000 individuals and contributing substantially to morbidity, mortality, and health care costs. Despite advances in therapies, HF care delivery remains fragmented, with significant variability in access to diagnostics, specialized services, and guideline-directed medical therapy (GDMT). Critically, the challenge is not a lack of evidence, but rather gaps in coordination, implementation, and accountability. Three dominant system-level issues drive poor outcomes: delayed diagnosis, vulnerability during transitions of care, and GDMT underuse. These are further compounded by geographic and sociodemographic disparities, particularly in rural, remote, and Indigenous communities, as well as the absence of standardized performance metrics and accountability frameworks. Evidence from national and international health care systems demonstrates that coordinated, data-driven approaches---incorporating standardized care pathways, performance measurement, and system-level accountability---can improve outcomes and efficiency. Early diagnosis, timely specialist follow-up, GDMT optimization, and multidisciplinary care models are associated with fewer hospitalizations, improved survival, and lower costs. Addressing these gaps will require a coordinated national strategy. Near-term priorities include establishing a national HF strategy, expanding access to diagnostic testing and improving GDMT access. Medium-term priorities include implementing standardized care pathways, strengthening care coordination, and enhancing data infrastructure to support performance measurement and quality improvement. Over the longer term, integrating HF into broader chronic disease frameworks and scaling digital solutions will be essential to ensure sustained coordination, accountability, and equitable access. Bill S-204 provides a critical opportunity to align key partners and advance a comprehensive, patient-centred, evidence-informed approach to HF care in Canada.

## Résumé

L'insuffisance cardiaque (IC) constitue un enjeu croissant de santé publique au Canada : elle touche plus de 787 000 personnes et contribue de manière significative à la morbidité, à la mortalité et aux coûts des soins de santé. Malgré les progrès thérapeutiques, la prise en charge de l'IC reste fragmentée, avec d'importantes disparités dans l'accès aux examens diagnostiques, aux services spécialisés et au traitement médical conforme aux lignes directrices (GDMT). Il est important de souligner que le problème ne réside pas dans un manque de données probantes, mais dans des lacunes en matière de coordination, de mise en œuvre et de responsabilisation. Trois grands enjeux systémiques contribuent aux mauvais résultats : les retards de diagnostic, la vulnérabilité lors des transitions de soins et la sous-utilisation du GDMT. Ces difficultés sont encore aggravées par des disparités géographiques et sociodémographiques, en particulier dans les communautés rurales, isolées et autochtones, ainsi que par l’absence d’indicateurs de performance standardisés et de cadres de responsabilisation.

Les données issues des systèmes de santé nationaux et internationaux démontrent que des approches coordonnées et fondées sur les données – intégrant des parcours de soins standardisés, une mesure de la performance et la responsabilisation à l’échelle du système – peuvent améliorer les résultats et l’efficience. Un diagnostic précoce, un suivi spécialisé en temps opportun, l’optimisation du GDMT et des modèles de soins multidisciplinaires sont associés à une diminution des hospitalisations, à une amélioration de la survie et à une réduction des coûts.

Pour combler ces lacunes, une stratégie nationale coordonnée sera nécessaire. Les priorités à court terme comprennent la mise en place d’une stratégie nationale sur l’IC, l’élargissement de l’accès aux tests diagnostiques et l’amélioration de l’accès au GDMT. Les priorités à moyen terme comprennent la mise en œuvre de parcours de soins standardisés, le renforcement de la coordination des soins et l’amélioration des infrastructures de données pour soutenir la mesure des performances et l’amélioration de la qualité. À plus long terme, l’intégration de l’IC dans des cadres plus larges de prise en charge des maladies chroniques et le déploiement à grande échelle de solutions numériques seront essentiels pour garantir une coordination durable, la responsabilisation et un accès équitable. Le projet de loi S-204 offre une occasion cruciale de mobiliser les principaux partenaires et de faire progresser une approche globale, centrée sur le patient et fondée sur des données probantes en matière de prise en charge de l’IC au Canada.

Heart failure (HF) is a common chronic condition for which effective therapies and models of care are increasingly available. Despite these advances, the delivery of HF care across Canada remains inconsistent. Patients frequently experience delays in diagnosis, uneven access to specialized services, and variability in the use of guideline-recommended therapies.^1^^,^^2^ Care pathways are often poorly integrated across settings, contributing to fragmentation, inefficiencies, and inequities in outcomes---particularly among rural, remote, and underserved populations.^3^

At its core, the challenge is not the absence of effective treatments or care models, but the difficulty of implementing them reliably and equitably at scale. This gap highlights a critical opportunity to improve outcomes through better alignment of systems, processes, and accountability mechanisms.

Three interrelated areas warrant focused attention: timely and accurate diagnosis, safe and coordinated transitions of care, and consistent initiation and rapid optimization of guideline-directed medical therapy (GDMT). Addressing these priorities will require coordinated strategies supported by measurable performance indicators and system-level oversight.^4^^,^^5^

In this article we examine the current state of HF care in Canada and outline practical, policy-relevant approaches to strengthen care delivery through coordinated, patient-centred, and evidence-informed strategies.

## What Is the Issue and Why Does it Matter?

HF is a growing global public health challenge and one of the most resource-intensive conditions worldwide. In Canada, more than 787,000 people are currently living with HF, with over 111,000 new diagnoses each year.^6^ Despite improvements in care, HF remains associated with substantial premature mortality, and nearly half of these patients die within 5 years of diagnosis if the disease is left untreated.^7^

The burden of HF extends beyond patients and caregivers to the health care system. HF is currently the third most common cause of hospitalization in Canada,^8^ and is associated with high rates of readmission, with 21% of patients readmitted within 30 days of discharge.^9^ Hospitalizations are the primary driver of HF-related costs, accounting for more than $6.65 billion in health care expenditures between 2010 and 2019, with costs projected to increase substantially over the coming decades.^10^

Taken together, the substantial clinical and economic burden of HF underscores the urgent need for strategies to improve prevention, diagnosis, treatment, and care delivery for Canadians living with HF.

## What Are the Barriers and Gaps?

Despite major advances in HF management, significant gaps persist across the Canadian health care system. Barriers occur at multiple levels, including limited access to diagnostic testing, inconsistent availability of specialized HF programs, delays in post-discharge follow-up, underuse of GDMT, fragmented care coordination, and systemic health inequities.^1^^,^^2^ National surveys suggest that these challenges are widespread and contribute to variation in the quality and accessibility of HF care across regions of the country.^1^^,^^2^

### Delayed diagnosis and limited early detection

Early and accurate diagnosis of HF is essential to initiate appropriate treatment and prevent disease progression. Measurement of natriuretic peptide levels is recommended in clinical guidelines to improve diagnostic accuracy and facilitate appropriate referral pathways.^11^^,^^12^ However, access to this testing remains inconsistent across Canada. In a national **H**eart **F**ailure **R**esources **a**nd **S**ervices **I**nventory (HF-RaSI) survey conducted between 2020 and 2021 across Canadian hospitals, 28.7% of centres reported no access to natriuretic peptide testing, with substantial provincial variation.^1^

Similarly, transthoracic echocardiography (TTE)---the cornerstone imaging modality for HF diagnosis---remains unavailable in many hospitals. The HF-RaSI survey found that only 48.1% of hospitals with HF admissions had onsite TTE, and 14.4% of centres had no access to TTE either onsite or through referral pathways.^1^ Even in regions where echocardiography is available, prolonged wait times may delay confirmation of diagnosis, initiation of evidence-based therapies, and referral to specialized services. These diagnostic gaps are particularly pronounced in smaller rural hospitals, where limited access to imaging infrastructure and trained personnel further restricts timely diagnosis.^1^

### Unequal access to specialized HF programs

Access to multidisciplinary HF clinics, which are associated with improved outcomes and reduced hospitalizations, remains uneven across Canada. Despite the fact that HF is the third most common admitting diagnosis, findings from the HF-RaSI survey revealed that 84% of hospitals lacked a dedicated inpatient HF team and 72% lacked a dedicated outpatient HF program.^1^ Overall, only 12.2% of HF care was delivered in centres with specialized HF expertise, whereas more than half of HF admissions occurred in hospitals with minimal HF resources.^1^

Contemporary data from Ontario further highlight the mismatch between capacity and demand.^13^ Despite a marked increase in patient volumes over the past decade, the number of HF clinics and providers has remained largely unchanged, with persistent variability in services offered across centers.^13^ These findings suggest that the existing HF clinic infrastructure has not kept pace with the growing burden of disease, contributing to constrained access and variability in care delivery.^13^

Even where HF clinics exist, variation in referral criteria and clinic capacity may limit access for many patients. Differences in patient selection practices, resource constraints, geographic disparities, and uncertainty regarding which patients derive the greatest benefit from specialized care contribute to heterogeneity in referral patterns and delays in accessing HF clinic services.^2^

### Variation in post-discharge care and clinic services

Timely follow-up after an HF hospitalization is critical to reducing readmissions. However, access to HF clinics after discharge varies substantially. Approximately half of HF clinics accept all patients discharged after HF hospitalization,^2^ and many centres report wait times of more than 2 weeks for an initial appointment, which exceeds recommended **C**anadian **C**ardiovascular **S**ociety (CCS) benchmarks.^1^ Importantly, early outpatient follow-up after discharge is associated with reduced readmissions and improved long-term prognosis, underscoring the clinical impact of these delays.^14^

HF clinics provide multidisciplinary services, including GDMT optimization, patient education, dietary counselling, and advance care planning.^2^ Although most clinics offer optimization of GDMT, fewer provide remote monitoring or comprehensive secondary prevention services, such as smoking cessation programs or cardiac rehabilitation.^2^ Where available, remote monitoring strategies are predominantly telephone-based, with relatively few clinics leveraging implantable device diagnostics or web- and app-based technologies.^2^

### Underuse of GDMT

Despite strong evidence supporting GDMT for HF, treatment gaps remain common. Data from the **C**anadian **H**eart **F**ailure (CAN-HF) Registry demonstrate that, although medication use increased during hospitalization for acute HF, important gaps persisted at discharge. Only 63% of patients received renin-angiotensin system inhibition, 80% received beta-blockers, and 40% received mineralocorticoid receptor antagonists.^15^ These therapies have consistently demonstrated mortality benefit in HF populations and are recommended as first-line treatments in national and international guidelines.^11^^,^^12^^,^^16^ More recently, underuse has also been observed with sodium-glucose cotransporter-2 inhibitors (SGLT2is). A population-based study in Alberta showed that only 10.2% of patients with diabetes and established HF were dispensed a SGLT2i, despite guideline recommendations, highlighting a significant treatment gap and the need for contemporary national data on SGLT2i implementation and uptake.^17^

Multiple factors contribute to GDMT underuse, including therapeutic inertia, concerns about adverse effects, polypharmacy, and restrictions in drug reimbursement policies.^9^^,^^18^, ^19^, ^20^ In several jurisdictions, reimbursement for medications, such as sacubitril-valsartan and SGLT2i, is subject to specific eligibility criteria, prior authorization requirements, or other formulary restrictions, which may delay or limit access to therapy. Variability in public drug coverage across provinces may further contribute to inequities in access to newer evidence-based therapies, including SGLT2i, despite strong evidence supporting their use across the spectrum of HF.^18^

### Fragmented care across the health system

Patients with HF frequently interact with multiple components of the health care system, including hospitals, primary care providers, specialists, and community services. Without thoughtful and deliberate care coordination, these transitions can result in fragmented care and poor patient experience.^2^ Survey data suggest that coordination between HF clinics and primary care providers remains limited. Less than half of HF clinics indicated that a majority of their patients had an identified primary care provider, and only 21% had established shared-care management strategies with primary care.^2^ In addition, only 24% of clinics indicated the existence of a provincial HF care pathway, highlighting the lack of standardized approaches to HF care delivery across jurisdictions.^2^

### Systemic health inequities

Significant disparities in HF care exist across geographic and demographic groups. Rural and remote communities face substantial barriers related to travel distance, limited health infrastructure, and shortages of specialized personnel.^1^ Many smaller provinces and territories lack cardiac centres capable of providing specialized interventions such as cardiac catheterization, cardiac surgery, or transplantation.^1^

Indigenous populations experience a disproportionate burden of cardiovascular disease, including HF, and are more likely to experience adverse outcomes.^3^^,^^21^, ^22^, ^23^, ^24^ These disparities reflect the complex interplay of geographic barriers, socioeconomic factors, systemic inequities, and the enduring impacts of colonialism within the health care system.^3^^,^^22^ Women also experience worse HF outcomes in Canada, with HF mortality approximately 20% higher among women than men.^21^^,^^25^

### Lack of performance targets and accountability

Finally, HF care in Canada lacks standardized performance measurement and accountability frameworks. Although the CCS has developed national quality indicators for HF care, implementation and routine measurement of these indicators remain inconsistent across jurisdictions.^26^ Key metrics that could be consistently measured include GDMT optimization, 30-day readmission rates, and patient-reported outcomes such as the **K**ansas **C**ity **C**ardiomyopathy **Q**uestionnaire (KCCQ).^27^ Moreover, patient engagement in quality improvement initiatives remains limited.^2^ Evidence suggests that systematic measurement and feedback of these performance metrics can improve care delivery and clinical outcomes.^27^

However, establishing quality indicators alone is insufficient without the infrastructure to support them. Many centres lack integrated data systems capable of capturing, extracting, linking, and reporting standardized metrics across care settings. Strengthening the data infrastructure---including interoperable electronic health records, standardized data definitions, linkage to administrative outcomes databases, and automated reporting tools---is essential to real-time performance tracking, benchmarking, and continuous improvement of HF care delivery across the country.

## What Can Be Learned From Other Jurisdictions?

Several provincial and national initiatives aimed at improving chronic disease management provide important lessons for strengthening HF care in Canada. These examples demonstrate how coordinated strategies that combine surveillance, performance measurement, and system-level accountability can improve care delivery and patient outcomes.

### National chronic disease frameworks

In Canada, diabetes provides a relevant example of how national policy can support improvements in chronic disease care. Similar to HF, diabetes is one of the most common chronic conditions affecting Canadians and its prevalence continues to rise globally.^28^ Recognizing this growing burden, the National Framework for Diabetes Act was passed in 2021, mandating the development of a national framework to improve diabetes prevention, treatment, and outcomes in Canada.^29^

The Framework for Diabetes in Canada outlines 6 interconnected areas where coordinated national action can advance care: prevention; management, treatment, and care; research; surveillance and data collection; learning and knowledge sharing; and access to devices, medications, and financial supports.^28^ Cross-cutting principles include addressing health equity, supporting innovation, promoting collaboration, and adopting a person-centred approach to care.^28^

These domains map directly onto key gaps in HF care. Enhanced surveillance and data collection could address the current lack of standardized performance measurement and accountability; improved access to medications and financial supports aligns with persistent barriers to GDMT optimization; and a stronger focus on equity could help mitigate geographic and sociodemographic disparities in HF care. In parallel, efforts to advance standardized care pathways, knowledge translation, and coordinated care models could improve timely diagnosis, ensure early post-discharge follow-up, and enhance integration across care settings.

Emerging provincial initiatives further demonstrate how these principles can be operationalized through digital infrastructure that embeds accountability into routine care. For example, Alberta’s Connect Care program integrates clinical decision support within the electronic health record, linking real-time clinical data with evidence-based guidelines to influence decision-making at the point of care.^30^

Although digital infrastructure can support standardized care delivery and accountability, coordinated organizational models are also needed to ensure that patients can access the right level of care at the right time. Ontario's Integrating Heart Failure Care initiative provides a Canadian example of a regionalized approach to HF care through a spoke-hub-node framework that links primary care providers, community services, multidisciplinary HF programs, and tertiary referral centres.^31^ By establishing standardized referral pathways and clearly defined roles across the continuum of care, this model aims to improve coordination, reduce variation in care delivery, and facilitate access to specialized services according to patient complexity.^31^ Such regionalized networks may provide a scalable framework for organizing HF care across Canada.

Taken together, these national and provincial initiatives demonstrate that meaningful improvements in chronic disease care require more than evidence-based therapies alone. Coordinated policy frameworks, robust data infrastructure, and integrated care delivery networks are all essential components of high-performing health systems. Given the substantial overlap between HF and other chronic conditions such as diabetes and chronic kidney disease, leveraging existing chronic disease frameworks and infrastructure offers an opportunity to align strategies, reduce duplication, and achieve economies of scale. Together, these elements provide a practical and scalable foundation for the development of a coordinated national HF strategy.

### National heart failure quality audits

Internationally, several countries have implemented national HF quality improvement programs that monitor care delivery and outcomes. The United Kingdom National Heart Failure Audit, administered by the **N**ational **I**nstitute for **C**ardiovascular **O**utcomes **R**esearch (NICOR), collects detailed data on HF hospitalizations across the National Health Service and provides hospitals with performance feedback to support quality improvement initiatives.^4^

The audit tracks key quality indicators linked to improved HF outcomes, including access to echocardiography, specialist HF care, GDMT optimization, and timely follow-up after hospital discharge. These data are used to monitor adherence to national clinical standards and inform health system planning.^4^ In addition, performance metrics are tied to the Best Practice Tariff, a financial incentive introduced in 2016 that rewards hospitals for meeting predefined HF quality targets.

Recent reports from the audit demonstrated high levels of adherence to GDMT, with 92% of patients receiving beta-blockers, 85% receiving angiotensin-converting enzyme inhibitors/angiotensin receptor neprilysin inhibitors/angiotensin receptor blockers, and 75% receiving SGLT2i in 2024-25.^32^ The audit has also shown that outcomes are improved when patients receive specialist HF care.^32^

### Quality improvement registries

The **G**et **W**ith **t**he **G**uidelines---**H**eart **F**ailure (GWTG-HF) program, launched by the American Heart Association in 2005, is a national quality improvement initiative designed to promote the implementation of evidence-based HF management in clinical practice.^5^ The program combines hospital-based registry data with education, benchmarking, and performance feedback on key performance indicators, including the use of diagnostic testing, prescription of GDMT, and discharge planning processes.^5^ Since its inception, the program has expanded to include more than 600 participating hospitals in the United States and has enabled linkage with Centers for Medicare and Medicaid Services data to evaluate longitudinal outcomes.^5^

Participation in the GWTG-HF program has been associated with improved adherence to evidence-based therapies and better process-of-care measures compared with non-participating hospitals.^33^ The registry also provides a platform for identifying care gaps, benchmarking institutional performance against peer institutions, and supporting implementation research aimed at improving HF care delivery.^5^^,^^34^ In addition, the program has helped define national quality metrics for HF management, which are regularly updated to reflect emerging evidence and clinical guidelines.^5^

Importantly, the GWTG-HF registry has also provided insights into how performance metrics linked to financial incentives or penalties may influence HF care delivery and outcomes.^5^ Analyses examining 30-day risk-adjusted readmission rates, which are used to determine financial penalties for hospitals under the US Hospital Readmissions Reduction Program, showed similar levels of adherence to key performance measure---including the prescription of GDMT---in hospitals with both high and low readmission rates.^35^ Moreover, although the implementation of financial penalties was associated with modest reductions in readmission rates, observational data suggest that mortality rates increased during the same period.^36^ These findings highlight the complexity of linking financial incentives or penalties to single performance indicators and underscore the importance of carefully designing quality improvement initiatives that prioritize patient outcomes alongside system performance.

Although the GWTG-HF program is voluntary and may not fully reflect the broader health system, it provides a compelling model for how national quality improvement initiatives can improve HF care through standardized measurement, feedback, and collaboration.

In contrast, Canada lacks a sustained, nationally coordinated HF quality improvement registry. Prior efforts, such as the CAN-HF Registry, have been limited in scope, with incomplete registration across provinces and major centres, and data collection confined to finite time periods.^15^ Given the rapid and ongoing evolution of HF care, there is a need for a contemporary, continuously updated national registry to support real-time monitoring, quality improvement, and system planning. Beyond data collections, challenges remain in accessing and linking clinical data across institutions and jurisdictions, including linkage to administrative databases that capture longitudinal outcomes such as mortality, hospitalizations, and health care use. Notably, Canada lags behind other countries that have established sustained, nationwide HF registries,^32^^,^^33^ further underscoring this important gap in infrastructure. Closing this gap is essential to advancing accountability, improving care delivery, and ensuring equitable outcomes for patients with HF across Canada.

## What Benefits Could Be Realized From Improvements?

Improving access to timely diagnosis, evidence-based therapies, and coordinated HF care has the potential to generate substantial benefits for patients, the health care system, and society.

### Improved diagnostic accuracy and system efficiency through earlier detection

Expanding access to natriuretic peptide testing improves diagnostic accuracy and supports timely initiation of evidence-based therapies, which can slow disease progression and improve long-term outcomes.^11^^,^^12^ In addition, broader implementation of N-terminal pro–B-type natriuretic peptide (NT-pro BNP) testing may improve health system efficiency by reducing unnecessary investigations and helping health care providers more rapidly distinguish HF from other causes of dyspnea, although the magnitude of these benefits will depend on implementation and health care use.^37^^,^^38^

### Reduced hospitalizations and mortality through coordinated, multidisciplinary care

Early follow-up after hospital discharge in a multidisciplinary HF clinic is well established to reduce readmissions, improve adherence to medical therapy, and enhance patient outcomes during vulnerable transitions of care.^39^ In a large Canadian cohort study by Lee et al., a structured approach incorporating early risk stratification and standardized transitional care resulted in a 12% reduction in all-cause mortality and cardiovascular hospitalizations compared with usual care.^40^ Similarly, care delivered by HF specialists and multidisciplinary teams has been associated with significant improvements in outcomes. In a large cohort of over 227,000 patients hospitalized with HF, specialist involvement during admission was associated with higher rates of GDMT, as well as lower in-hospital and long-term mortality.^41^ These benefits were particularly pronounced among patients with heart failure with reduced ejection fraction, likely reflecting more rapid initiation and optimization of disease-modifying therapies.^41^ Leveraging multidisciplinary team members, including pharmacist-led medication optimization clinics established in several Canadian centres, may further improve GDMT uptake and has been associated with reduced in hospitalizations and mortality.^42^ Findings are consistent with evidence from the **S**afety, **T**olerability, and Efficacy of **R**apid **O**ptimization, Helped by **N**T-pro BNP Testin**g**, of **H**eart **F**ailure Therapies (STRONG-HF) trial, which demonstrated that early and intensive optimization of GDMT reduces the risk of mortality and HF rehospitalization without an increase in adverse events.^39^

### Improved survival and cost savings through optimization of medical therapy

Improving access to and uptake of GDMT represents another major opportunity to improve outcomes and reduce health care costs. A Canadian population-based analysis estimated that optimal use of sacubitril-valsartan could result in a 15% reduction in HF-related mortality and generate more than $40 million in annual savings through reductions in HF hospitalizations.^43^ These findings highlight the potential impact of addressing reimbursement barriers, even for generic medications, and supporting broader implementation of evidence-based therapies.

### Improved care quality and outcomes through quality improvement initiatives

Large-scale quality assurance and improvement programs have demonstrated that systematic approaches to HF care can improve adherence to evidence-based therapies and clinical outcomes. For example, hospitals participating in the GTWG-HF program have shown improved documentation of left ventricular ejection fraction (93.4% vs 88.8%; *P* < 0.001), higher rates of GDMT use (use of angiotensin-converting enzyme inhibitors or angiotensin receptor blockers: 88.3% vs 86.6%; *P* = 0.03), and modest reductions in readmission rates (24.2% vs 24.6%; *P* < 0.001) compared with non-participating hospitals.^5^ Higher functioning hospitals were associated with improved 1-, 3-, and 5-year survival among patients with HF.^44^ These findings suggest that the GTWG-HF program may contribute to improved care quality, even after adjustment for differences in hospital characteristics.^5^

In addition, these programs have played an important role in identifying and addressing care gaps. Early analyses from GTWG-HF highlighted low uptake of angiotensin receptor neprilysin inhibitors after their introduction (2.3% of eligible patients), with subsequent improvements over time to 55.4% in eligible patients, as awareness and implementation increased.^45^^,^^46^ The registry has also been instrumental in identifying disparities in care, including lower rates of implantable cardiac device therapy among women and racialized populations, prompting targeted quality improvement efforts.^47^, ^48^, ^49^

### Reduced health care use and costs through remote monitoring and community-based care

Emerging evidence supports the role of remote monitoring and community-based care models in reducing health care use. Remote monitoring typically involves the collection of patient data (eg, symptoms, weight, and vital signs), whereas remote management incorporates structured clinical review and timely intervention based on these data. International studies have demonstrated that multidisciplinary care models incorporating remote management, which combines home- or telephone-based monitoring with structured clinical review and escalation, can reduce emergency department visits by up to 75%, HF-related readmissions by up to 89%, and health care costs by 63% compared with usual care, with an estimated cost savings of US $4583 per individual after hospital discharge.^50^ Data from British Columbia similarly demonstrate cost savings associated with home telemonitoring in patients with HF.^51^ However, the magnitude of these benefits remains uncertain as many studies were limited by pre-post study designs and potential selection bias. Furthermore, randomized trials of noninvasive remote monitoring strategies have yielded mixed results,^52^ whereas the most consistent evidence of reductions in HF hospitalizations has been observed with implantable hemodynamic monitoring systems.^53^^,^^54^ Despite these limitations, coordinated, technology-enabled models of care have the potential to improve efficiency and reduce avoidable health care use when integrated within broader multidisciplinary HF care programs. Beyond reducing hospitalizations, coordinated HF care may also improve the appropriateness of health care use by reducing unnecessary repeat investigations and facilitating earlier identification of clinical deterioration, allowing timely intervention before recurrent decompensation or hospitalization occurs.

### Improved quality of life and reduced caregiver burden with broader societal benefits

Beyond reducing mortality and hospitalizations, improving access to evidence-based HF therapies and coordinated care can substantially improve patient quality of life. In the STRONG-HF trial, rapid initiation and optimization of GDMT was associated with clinically meaningful improvements in health-related quality of life, symptoms, and functional status compared with usual care.^39^ These findings highlight that the benefits of timely implementation of GDMT extend beyond traditional clinical outcomes and include improvements that are directly meaningful to patients.

HF is also associated with a substantial psychosocial burden, including depression, anxiety, social isolation, and reduced quality of life. Investments in comprehensive HF care that incorporate psychosocial support, patient education, and self-management resources may further enhance patient well-being and engagement in care.^55^

Importantly, the impact of HF extends beyond the individual patient to families and caregivers. Suboptimal disease management places a significant burden on caregivers, contributing to emotional stress, financial strain, and reduced quality of life.^56^ Improving access to comprehensive, coordinated HF care can help alleviate this burden by supporting patient self-management, reducing acute care needs, and enhancing overall stability.^56^

Taken together, these findings suggest that improving HF care delivery could simultaneously reduce mortality, decrease hospitalizations, improve patient and caregiver quality of life, and generate substantial cost savings for the Canadian health care system.

## What Actions Are Needed to Support Improvement?

Improving HF care in Canada will require coordinated action across health systems, governments, and stakeholders (Fig. 1). The Heart & Stroke Foundation HF Action Plan Advisory Committee has outlined key priorities to strengthen HF care delivery, emphasizing integrated systems, equitable access, data-driven quality improvement, and patient-centred care.^21^ Building on these priorities, several short-, medium-, and long-term actions can support meaningful and sustained improvements in HF care across Canada (Fig. 2).

**Figure 1:**
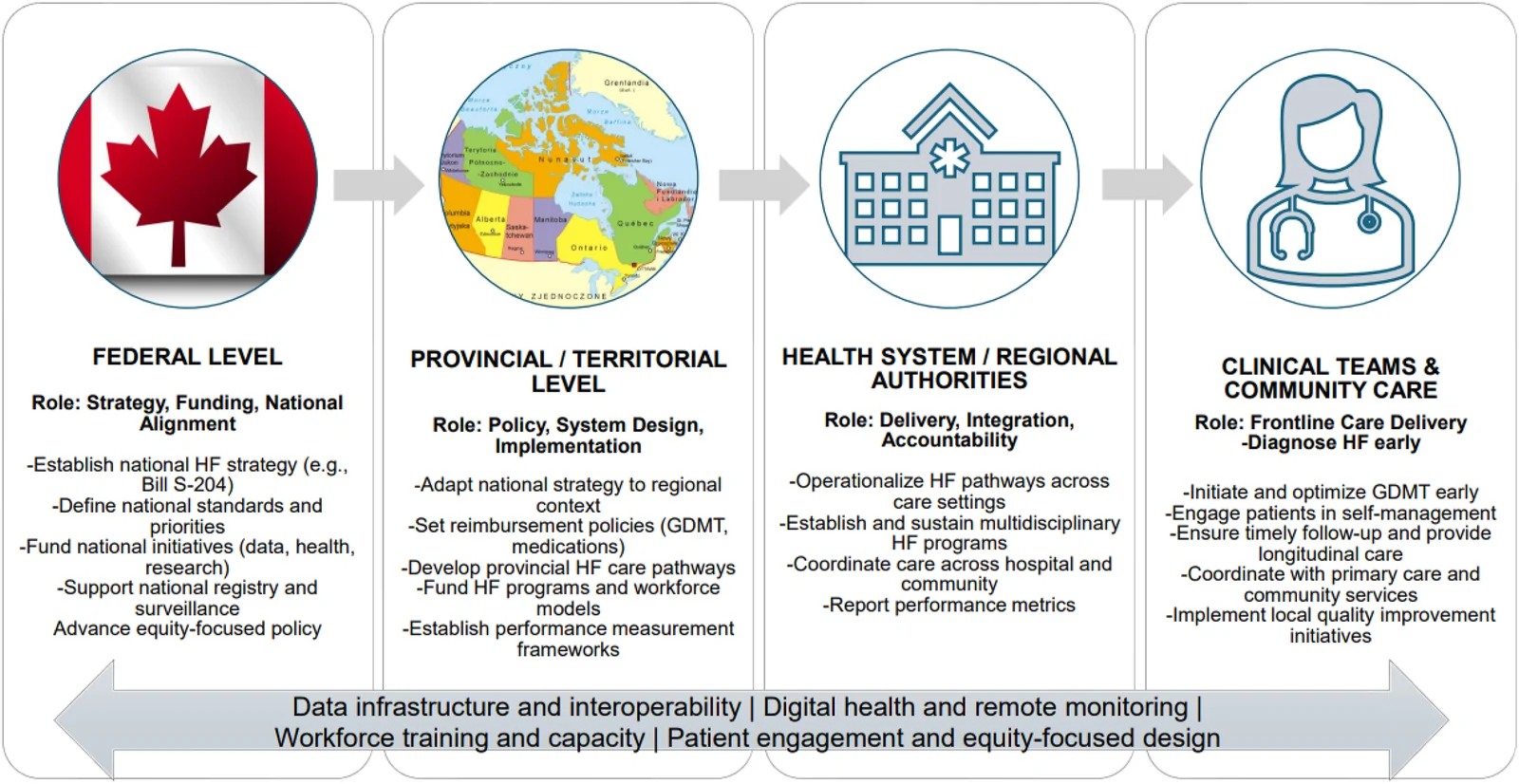
Governance framework for operationalizing a national heart failure strategy in Canada. GDMT, guideline-directed medical therapies; HF, heart failure.

**Figure 2:**
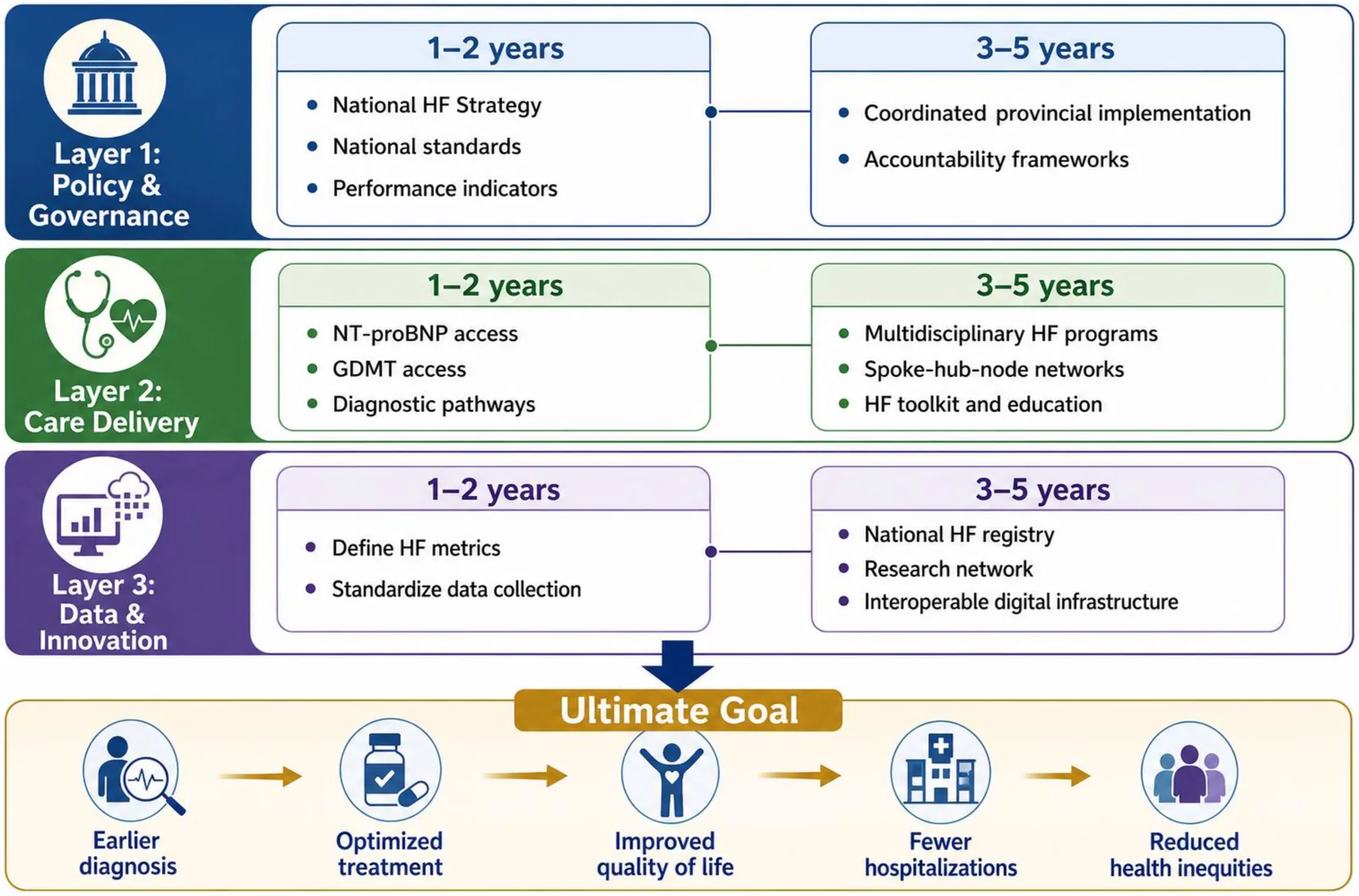
Roadmap for implementation of a national heart failure strategy in Canada. HF, heart failure; GDMT, guideline-directed medical therapies; NT-proBNP: N-terminal pro–B-type natriuretic peptide.

### Short-term actions (1-2 years)

#### Develop a national HF strategy

A coordinated Canadian heart failure strategy, such as that proposed in Bill S-204, would provide a national framework to improve prevention, diagnosis, treatment, and care delivery.^57^ Key elements should include:

• Promoting early detection and equitable access to care.
• Expanding multidisciplinary and technology-enabled care models.
• Addressing disparities in rural, remote, and underserved populations.
• Establishing national data infrastructure and performance indicators.

The recent introduction of Bill S-204 has increased national attention on HF and may provide a mechanism to advance many of these priorities. By bringing together key stakeholders to develop a coordinated framework for HF care, it could help align federal and provincial efforts, establish shared priorities, and support the implementation of a national HF strategy.

Successful implementation will require collaboration among patients, clinicians, professional societies, health system leaders, as well as provincial and federal governments. Potential barriers include fragmented jurisdictional responsibilities, competing health care priorities, variability in provincial resources, and absence of dedicated funding and accountability mechanisms to support implementation.

#### Improve access to diagnostics and evidence-based therapies

Improving access to diagnostic testing and GDMT is essential. Access to natriuretic peptide testing and echocardiography remains inconsistent across Canada and should be expanded through strategies such as point-of-care testing, telehealth-enabled interpretation, and workforce training in underserved regions.^1^^,^^2^^,^^58^

In parallel, policy efforts should focus on modernizing outdated drug reimbursement criteria and reducing financial barriers to GDMT, ensuring equitable access across jurisdictions in alignment with the latest CCS HF guidelines.^18^^,^^38^

Achieving these goals will require coordination among governments, payers, health care organizations, and professional societies. Key barriers include regional variation in reimbursement policies, workforce shortages, limited diagnostic infrastructure in some communities, and disparities in access to specialized services across rural and remote regions.

### Medium-term actions (3-5 years)

#### Strengthen HF care delivery and coordination

A thoughtful health human resources strategy is essential to support the delivery of HF care. Despite increasing demand, there are ongoing challenges in recruiting and retaining clinicians across disciplines involved in HF management. In particular, relatively few cardiologists are pursuing specialization in HF, and limited incentives exist to support multidisciplinary team–based care.^59^ Prior work by the Canadian Heart Failure Society has highlighted gaps in workforce capacity and variability in team composition across the country.^59^ Addressing these challenges will require targeted workforce planning, training pathways, and funding models that incentivize team-based, longitudinal HF care.^59^

Central to a coordinated national HF strategy is the establishment and support of multidisciplinary team–based models of care. Effective HF management requires collaboration among cardiologists, primary care providers, nurses, nurse practitioners, physician assistants, pharmacists, dietitians, social workers, rehabilitation specialists, palliative care providers, and community-based health care professionals. Given the growing prevalence of HF and recognized workforce shortages, scalable models of care must empower non-specialist providers to manage patients with less complex HF closer to home, while reserving specialized HF resources for patients with greater complexity. Regionalized care frameworks, such as spoke-hub-node models, can support this approach by facilitating collaboration across levels of care and providing access to specialist consultation when needed. Leveraging the expertise of multidisciplinary team members can improve GDMT optimization, care coordination, patient education, self-management, and transitions of care while helping address workforce limitations and geographic disparities in access to specialized services.

Building on this foundation, standardized clinical protocols, including admission order sets, discharge pathways, referral processes, clinical decision support tools, and educational resources, can further support consistent and high-quality care delivery. This includes developing regional and provincial care pathways, implementing coordinated referral systems with defined timelines, expanding access to multidisciplinary teams, and supporting primary care and other non-specialist health care providers in delivering evidence-based HF care.^2^^,^^38^ There is also a need to address variability in educational care models through the development of a nationally curated HF toolkit, ensuring consistency and alignment with evidence-based practice.

Care delivery models, educational resources, and clinical tools should be co-created using a person-centred and culturally appropriate approach, particularly for Indigenous and underserved populations, incorporating principles of cultural safety and engagement with community-based models of care.^3^^,^^60^ A strong emphasis on equity is essential in the design and implementation of these models. Tailored approaches are needed to address the unique challenges faced by rural, remote, and Indigenous communities, including geographic barriers, limited access to specialized services, as well as historical and ongoing systemic inequities. Strategies such as virtual care, community-based models, and partnerships with local providers can help improve access to culturally appropriate, accessible, and sustainable HF care while reducing disparities in outcomes across Canada.^61^, ^62^, ^63^

Successful implementation of these initiatives will require sustained investment, cross-sector collaboration, and alignment among health care organizations, professional societies, governments, and patient partners. Key barriers include workforce shortages, limited funding for multidisciplinary care models, variability in local infrastructure and resources, and challenges associated with coordinating care across multiple providers and jurisdictions. Addressing these barriers will require clear accountability structures, dedicated implementation funding, and ongoing evaluation to ensure that care models remain scalable, equitable, and responsive to local needs.

#### Support research and innovation

Ongoing investment in research is essential to address knowledge gaps and improve HF care delivery. National initiatives, such as the Canadian Heart Failure Research Network, can support collaboration among researchers, clinicians, patients, and policymakers.^21^ Future research should prioritize health equity, intersectionality, and patient engagement, ensuring that care strategies reflect the needs of diverse populations and address disparities in HF outcomes.^3^^,^^5^^,^^63^

Achieving these goals will require sustained investment in research infrastructure, data-sharing capabilities, and collaborative networks that bring together researchers, clinicians, patients, and policymakers. Key barriers include limited funding, fragmented research efforts, and challenges ensuring equitable representation of diverse populations in HF research.

### Enhance data infrastructure and performance measurement

Robust data infrastructure is critical to enabling quality improvement. There remains significant variability in the ability to measure clinical outcomes and processes of care across Canada.^2^ Key actions include defining national HF performance indicators, mandating routine performance reporting, and incorporating patient-reported outcome and experience measures into clinical practice and system evaluation.^38^ Improved integration of inpatient and outpatient data systems will enable benchmarking, monitoring of quality indicators, and more effective health system planning.^2^

Beyond performance measurement, strengthened data infrastructure is also essential to improving our understanding of HF in Canada. Comprehensive, longitudinal data can help characterize patient populations, identify key risk factors and comorbid conditions, and better define patterns of disease progression and care use. These insights are critical to informing upstream prevention strategies, enabling earlier identification of at-risk individuals and supporting more proactive, population-based approaches to care.

In this context, data systems should be designed not only to evaluate care delivery, but also to support a more holistic model of care that integrates clinical, demographic, and social determinants of health, with the goal of improving outcomes at both the individual and health system level.

Achieving these goals will require investment in interoperable digital infrastructure, standardized data definitions, and mechanisms to support data sharing across health care settings and jurisdictions. Key barriers include fragmented electronic health record systems; limited interoperability between inpatient and outpatient platforms; variability in data collection practices; and the resources required to support data extraction, reporting, and quality improvement activities. Overcoming these challenges will be essential to enabling meaningful performance measurement, benchmarking, and continuous improvement in HF care across Canada.

### Long-term actions (5-10 years)

#### Integrate HF into chronic disease frameworks

HF should be integrated into broader chronic disease strategies, with emphasis on multidisciplinary, patient-centred care models that include mental health and psychosocial support.^38^ Expanding access to mental health services, including counselling, peer support, and community-based resources, is particularly important given the significant psychosocial burden of HF.^38^

Successful integration of HF into broader chronic disease frameworks will require alignment across disease-specific programs, health care organizations, and government stakeholders to ensure that care models, quality indicators, and funding structures support coordinated management of multimorbidity. Key barriers include the traditional siloing of chronic disease programs, competing health care priorities, and limited integration of mental health and psychosocial services within existing cardiovascular care pathways. Addressing these challenges will be essential to delivering more holistic, person-centred care for individuals living with HF.

### Scale digital health and remote monitoring

Expanding the use of virtual care, remote monitoring, and digital health platforms can improve access to care and reduce geographic disparities. Investments in a unified and interoperable digital health ecosystem---including telehealth infrastructure, electronic medical records, and clinical decision support systems at point of care---are needed to enable seamless access to patient information across jurisdictions, improve care coordination, and support earlier diagnosis and intervention.^1^^,^^38^^,^^61^^,^^62^ The development of a national HF registry and strengthened data infrastructure will further support surveillance, resource planning, and evaluation of care delivery, allowing us to make more informed choices around health care investments in the future.^38^

Achieving these goals will require sustained investment in the digital infrastructure, interoperability standards, broadband connectivity, and workforce training to support the adoption of virtual care and remote monitoring technologies. Key barriers include fragmented electronic health record systems; variability in digital health infrastructure and capabilities across jurisdictions; limited operability between health care platforms; and disparities in access to technology among rural, remote, and underserved populations. Addressing these challenges will be critical to ensuring that digital health solutions improve equity and care coordination rather than inadvertently widening existing gaps in access to care. Taken together, these actions highlight the need for a coordinated, data-driven, patient-centred approach to HF care, with the potential to improve outcomes, reduce disparities, and enhance the sustainability of Canada’s health care system.

## Conclusions

Heart failure represents a major and growing challenge for the Canadian health care system---but it is also an area where meaningful improvement is within reach. The evidence is clear: earlier diagnosis, consistent implementation of evidence-based therapies, and coordinated data-driven models of care can significantly improve outcomes while reducing the strain on our health care system.

Achieving this will require collaboration across governments, health system partners (eg, CCS), care providers, and patient partners, supported by a strengthened data infrastructure, aligned policy, and sustained commitment. Bill S-204 lays the groundwork for a national HF strategy; by working together to bring this vision to life, we have a transformative opportunity to improve care, outcomes, and equity for all Canadians living with HF in the years ahead.

## Acknowledgements

The authors thank the members of the Canadian Cardiovascular Society Advocacy Working Group and Heart Failure Working Group for their valuable input and feedback, as well as the colleagues who provided comments and suggestions during its development.

## Ethics Statement

The research reported adhered to relevant institutional and national ethical guidelines. Research ethics board approval was not required, as this work did not involve human participants or identifiable patient data.

## Patient Consent

The authors confirm that patient consent is not applicable to this article because this work is a narrative policy review and advocacy article and did not involve collection, analysis, or reporting of data from individual patients.

## Funding Sources

The authors have no funding sources to declare.

## Disclosures

The authors have no conflicts of interest to disclose.

## References

[1] Moghaddam N., Lindsay M.P., Hawkins N.M. (2023). Access to heart failure services in Canada: findings of the Heart and Stroke National Heart Failure Resources and Services Inventory. Can J Cardiol.

[2] Virani S.A., Zieroth S., Bray S. (2020). The status of specialized ambulatory heart failure care in Canada: a joint Canadian Heart Failure Society and Canadian Cardiovascular Society Heart Failure Guidelines Survey. CJC Open.

[3] Vervoort D., Kimmaliardjuk D.M., Ross H.J. (2022). Access to cardiovascular care for Indigenous peoples in Canada: a rapid review. CJC Open.

[4] Healthcare Quality Improvement Partnership National Heart Failure Audit (NHFA) 2021 summary report. https://www.hqip.org.uk/resource/national-heart-failure-audit-nhfa-2021-summary-report/#:∼:text=There%20were%2069%2C556%20admissions%20captured,test%20(now%20at%2086%25).

[5] Tang A.B., Lewsey S.C., Yancy C.W. (2025). Get With the Guidelines---Heart Failure: twenty years in review, lessons learned, and the road ahead. Circ Heart Fail.

[6] Health Canada Canadian chronic disease surveillance system. https://health-infobase.canada.ca/ccdss/data-tool.

[7] Roger V.L. (2013). Epidemiology of heart failure. Circ Res.

[8] Canadian Institute for Health Information Hospital stays in Canada, 2022-2023. https://www.cihi.ca/en/hospital-stays-in-canada-2022-2023.

[9] Poon S., Leis B., Lambert L. (2022). The state of heart failure care in Canada: minimal improvement in readmissions over time despite an increased number of evidence-based therapies. CJC Open.

[10] Ellis E.E., Quereshi H., Dover D.C. (2024). Economic burden of heart failure hospitalizations in Canada: a population-based study. CJC Open.

[11] Ezekowitz J.A., O'Meara E., McDonald M.A. (2017). 2017 Comprehensive update of the Canadian Cardiovascular Society Guidelines for the management of heart failure. Can J Cardiol.

[12] McDonald M., Virani S., Chan M. (2021). CCS/CHFS heart failure guidelines update: defining a new pharmacologic standard of care for heart failure with reduced ejection fraction. Can J Cardiol.

[13] Kugathasan L., Francis T., Rac V.E. (2021). A 2020 environmental scan of heart failure clinics in Ontario. CJC Open.

[14] Matsukawa R., Masuda S., Matsuura H. (2021). Early follow-up at outpatient care after discharge improves long-term heart failure readmission rate and prognosis. ESC Heart Fail.

[15] Poon S., Rojas-Fernandez C., Virani S. (2022). The Canadian Heart Failure (CAN-HF) registry: a Canadian multicentre, retrospective study of inpatients with heart failure. CJC Open.

[16] Heidenreich P.A., Bozkurt B., Aguilar D. (2022). 2022 AHA/ACC/HFSA guideline for the management of heart failure: executive summary: a report of the American College of Cardiology/American Heart Association joint committee on clinical practice guidelines. Circulation.

[17] Butalia S., Wen C., Sigal R. (2024). Real-world use and outcomes of sodium-glucose cotransporter-2 inhibitors in adults with diabetes and heart failure: a population-level cohort study in Alberta, Canada. Can J Diabetes.

[18] Cowan S.S., Kosar L., Poon S. (2025). Prescription drug coverage of guideline-directed medical therapy for people living with heart failure with reduced ejection fraction in Canada. CJC Open.

[19] Greene S.J., Khan M.S., Butler J. (2024). Why do clinicians not prescribe quadruple medical therapy for heart failure with reduced ejection fraction?. Eur J Heart Fail.

[20] Jarjour M., Henri C., de Denus S. (2020). Care gaps in adherence to heart failure guidelines: clinical inertia or physiological limitations?. JACC Heart Fail.

[21] Heart and Stroke Foundation of Canada Falling short: how Canada is failing people with heart failure---and how we can change that. https://www.heartandstroke.ca/-/media/pdf-files/canada/2022-heart-month/hs-heart-failure-report-2022-final.pdf?rev=245159ea1726419aaa6f71ae9e7692f3.

[22] Anand S.S., Abonyi S., Arbour L. (2019). Explaining the variability in cardiovascular risk factors among First Nations communities in Canada: a population-based study. Lancet Planet Health.

[23] Schultz A., Dahl L., McGibbon E. (2020). Differences in coronary artery disease complexity and associations with mortality and hospital admissions among First Nations and non-First Nations patients undergoing angiography: a comparative retrospective matched cohort study. CMAJ Open.

[24] Atzema C., Kapral M., Klein-Geltink J. Cardiovascular disease in the Métis Nation of Ontario: technical report. https://www.metisnation.org/wp-content/uploads/2020/04/81920final20cvd20technical20en.pdf.

[25] Keshvani N., Shah S., Ayodele I. (2023). Sex differences in long-term outcomes following acute heart failure hospitalization: findings from the Get With the Guidelines---Heart Failure registry. Eur J Heart Fail.

[26] McKelvie R.S., Heckman G.A., Blais C. (2016). Canadian Cardiovascular Society quality indicators for heart failure. Can J Cardiol.

[27] Schjødt I., Johnsen S.P., Strömberg A. (2022). Evidence-based process performance measures and clinical outcomes in patients with incident heart failure with reduced ejection fraction: a Danish nationwide cohort study. Circ Cardiovasc Qual Outcomes.

[28] Public Health Agency of Canada Framework for diabetes in Canada. https://www.canada.ca/content/dam/phac-aspc/documents/services/publications/diseases-conditions/framework-diabetes-canada/framework-diabetes-canada.pdf.

[29] Government of Canada (2021). National Framework for Diabetes Act. https://laws-lois.justice.gc.ca/eng/acts/n-9.1/fulltext.html.

[30] Alberta Health Services (2024). Connect Care. https://www.albertahealthservices.ca/cis/cis.aspx.

[31] Huitema A.A., Harkness K., Heckman G.A. (2018). The spoke-hub-and-node model of integrated heart failure care. Can J Cardiol.

[32] National Institute for Cardiovascular Outcomes Research (2024/25). https://www.nicor.org.uk/%7Edocuments/route%3A/download/3758.

[33] Heidenreich P.A., Hernandez A.F., Yancy C.W. (2012). Get With the Guidelines program participation, process of care, and outcome for Medicare patients hospitalized with heart failure. Circ Cardiovasc Qual Outcomes.

[34] Heidenreich P.A., Zhao X., Hernandez A.F. (2012). Patient and hospital characteristics associated with traditional measures of inpatient quality of care for patients with heart failure. Am Heart J.

[35] Pandey A., Golwala H., Xu H. (2016). Association of 30-day readmission metric for heart failure under the hospital readmissions reduction program with quality of care and outcomes. JACC Heart Fail.

[36] Gupta A., Allen L.A., Bhatt D.L. (2018). Association of the hospital readmissions reduction program implementation with readmission and mortality outcomes in heart failure. JAMA Cardiol.

[37] Siebert U., Milev S., Zou D. (2021). Economic evaluation of an N-terminal pro B-type natriuretic peptide-supported diagnostic strategy among dyspneic patients suspected of acute heart failure in the emergency department. Am J Cardiol.

[38] HeartLife Foundation (2024). Heart failure policy framework: a national action plan for change. https://heartlife.com/wp-content/uploads/2024/01/hl_policyframeworkreport.pdf#:%7E:text=these%20funding%20recommendations%20aim%20to,and%20accurate%20diagnosis%2C%20access%20to.

[39] Mebazaa A., Davison B., Chioncel O. (2022). Safety, tolerability and efficacy of up-titration of guideline-directed medical therapies for acute heart failure (STRONG-HF): a multinational, open-label, randomised, trial. Lancet.

[40] Lee D.S., Straus S.E., Farkouh M.E. (2023). Trial of an intervention to improve acute heart failure outcomes. N Engl J Med.

[41] Cannata A., Mizani M.A., Bromage D.I. (2025). Heart failure specialist care and long-term outcomes for patients admitted with acute heart failure. JACC Heart Fail.

[42] Cao V.F.S., Cowley E., Koshman S.L. (2021). Pharmacist-led optimization of heart failure medications: a systematic review. J Am Coll Clin Pharm.

[43] Huitema A.A., Daoust A., Anderson K. (2020). Optimal usage of sacubitril/valsartan for the treatment of heart failure: the importance of optimizing heart failure care in Canada. CJC Open.

[44] Pandey A., Patel K.V., Liang L. (2018). Association of hospital performance based on 30-day risk-standardized mortality rate with long-term survival after heart failure hospitalization: an analysis of the Get With the Guidelines---Heart Failure Registry. JAMA Cardiol.

[45] Luo N., Fonarow G.C., Lippmann S.J. (2017). Early adoption of sacubitril/valsartan for patients with heart failure with reduced ejection fraction: insights from Get With the Guidelines---Heart Failure (GWTG-HF). JACC Heart Fail.

[46] Srivastava P.K., Klomhaus A.M., Greene S.J. (2025). Angiotensin receptor-neprilysin inhibitor prescribing patterns in patients hospitalized for heart failure. JAMA Cardiol.

[47] Hernandez A.F., Fonarow G.C., Liang L. (2007). Sex and racial differences in the use of implantable cardioverter-defibrillators among patients hospitalized with heart failure. JAMA.

[48] Ellrodt A.G., Fonarow G.C., Schwamm L.H. (2013). Synthesizing lessons learned from Get With the Guidelines: the value of disease-based registries in improving quality and outcomes. Circulation.

[49] Al-Khatib S.M., Hellkamp A.S., Hernandez A.F. (2012). Trends in use of implantable cardioverter-defibrillator therapy among patients hospitalized for heart failure: have the previously observed sex and racial disparities changed over time?. Circulation.

[50] Vohra A.S., Chua R.F.M., Besser S.A. (2020). Community health workers reduce rehospitalizations and emergency department visits for low-socioeconomic urban patients with heart failure. Crit Pathw Cardiol.

[51] Apantaku G., Mitton C., Wong H. (2022). Home telemonitoring technology for patients with heart failure: cost-consequence analysis of a pilot study. JMIR Form Res.

[52] Inglis S.C., Clark R.A., Dierckx R. (2015). Structured telephone support or non-invasive telemonitoring for patients with heart failure. Cochrane Database Syst Rev.

[53] Abraham W.T., Adamson P.B., Bourge R.C. (2011). Wireless pulmonary artery haemodynamic monitoring in chronic heart failure: a randomised controlled trial. Lancet.

[54] Shavelle D.M., Desai A.S., Abraham W.T. (2020). Lower rates of heart failure and all-cause hospitalizations during pulmonary artery pressure-guided therapy for ambulatory heart failure: one-year outcomes from the CardioMEMS post-approval study. Circ Heart Fail.

[55] The Lancet Global Health (2020). Mental health matters. Lancet Glob Health.

[56] Suksatan W., Tankumpuan T., Davidson P.M. (2022). Heart failure caregiver burden and outcomes: a systematic review. J Prim Care Commun Health.

[57] Senate of Canada (2025). Bill S-204: an act to establish a national framework on heart failure. https://www.parl.ca/documentviewer/en/45-1/bill/S-204/first-reading.

[58] Tromp J., Seekings P.J., Hung C.L. (2022). Automated interpretation of systolic and diastolic function on the echocardiogram: a multicohort study. Lancet Digit Health.

[59] Almufleh A., Brahmbhatt D.H., Billia F. (2026). The Canadian Heart Failure Society (CHFS) Workforce Committee Report 2024: Addressing the challenges facing the heart failure physicians workforce in Canada CHFS 2024 heart failure workforce report. J Card Fail.

[60] Rieger K.L., Bennett M., Martin D. (2021). Digital storytelling as a patient engagement and research approach with First Nations women: how the medicine wheel guided our Debwewin journey. Qual Health Res.

[61] Kitsiou S., Vatani H., Paré G. (2021). Effectiveness of mobile health technology interventions for patients with heart failure: systematic review and meta-analysis. Can J Cardiol.

[62] Kuan P.X., Chan W.K., Fern Ying D.K. (2022). Efficacy of telemedicine for the management of cardiovascular disease: a systematic review and meta-analysis. Lancet Digit Health.

[63] Allana S., Ski C.F., Thompson D.R. (2021). Bringing intersectionality to cardiovascular health research in Canada. CJC Open.

